# Dual-energy CT quantification of extracellular liver volume predicts short-term disease progression in patients with hepatitis B liver cirrhosis-acute decompensation

**DOI:** 10.1186/s13244-023-01393-x

**Published:** 2023-03-29

**Authors:** Yuan Xu, Yufeng Li, Shenglin Li, Shouxiao Xue, Jianli Liu

**Affiliations:** 1 Department of Radiology, Lanzhou University Second Hospital, Lanzhou, China; 2 Second Clinical School, Lanzhou University, Lanzhou, China; 3 Key Laboratory of Medical Imaging of Gansu Province, Lanzhou University Second Hospital, Lanzhou, China

**Keywords:** Dual-energy CT, Extracellular liver volume, Liver cirrhosis-acute decompensation, Acute-on-chronic liver failure, Nomogram

## Abstract

**Background:**

Liver cirrhosis-acute decompensation (LC-AD) has rapid short-term disease progression and difficult early risk stratification. The purpose is to develop and validate a model based on dual-energy CT quantification of extracellular liver volume (ECV_IC-liver_) for predicting the occurrence of acute-on-chronic liver failure (ACLF) within 90 days in patients with hepatitis B (HBV) LC-AD.

**Methods:**

The retrospective study included patients with HBV LC-AD who underwent dual-energy CT scans of the liver from January 2018 to March 2022 and were randomized to training group (215 patients) and validation group (92 patients). The primary outcome was the need for readmission within 90 days due to ACLF. Based on the training group data, independent risk factors for disease progression in clinical and dual-energy CT parameters were identified and modeled by logistic regression analysis. Based on the training and validation groups data, receiver operating characteristic (ROC) curves, calibration curves, and decision analysis curves (DCA) were used to verify the discrimination, calibration, and clinical validity of the nomogram.

**Results:**

Chronic liver failure consortium-acute decompensation score (CLIF-C ADs) (*p* = 0.008) and ECV_IC-liver_ (*p* < 0.001) were independent risk factors for ACLF within 90 days. The AUC of the model combined ECV_IC-liver_ and CLIF-C ADs were 0.893 and 0.838 in the training and validation groups, respectively. The calibration curves show good agreement between predicted and actual risks. The DCA indicates that the model has good clinical application.

**Conclusion:**

The model combined ECV_IC-liver_ and CLIF-C ADs can early predict the occurrence of ACLF within 90 days in HBV LC-AD patients.

**Supplementary Information:**

The online version contains supplementary material available at 10.1186/s13244-023-01393-x.

## Key points

- Chronic liver failure consortium-acute decompensation score (CLIF-C ADs) and extracellular liver volume (ECV_IC-liver_) are independent risk factors for disease progression.
- Patients with a lower ECV_IC-liver_ have a better prognosis.
- Highest predictive efficacy using the model combining CLIF-C ADs and ECV_IC-liver_.

**Supplementary Information:**

The online version contains supplementary material available at 10.1186/s13244-023-01393-x.

## Background

Liver cirrhosis-acute decompensation (LC-AD) refers to the first occurrence or acute development of grade 2 or 3 ascites, hepatic encephalopathy, gastrointestinal bleeding, and infection within 2 weeks in patients with cirrhosis, and it is the most important variable for stratifying the risk of death in patients with cirrhosis [1, 2]. Three clinical courses with significant prognostic differences are observed in patients with LC-AD, some of whom will develop acute-on-chronic liver failure (ACLF) within 90 days, which is characterized by a severe systemic inflammatory response, multisystem/multiorgan failure, and high short-term mortality [3]. In 2020, the PREDICT study conducted by the European Association for the study of the liver-chronic liver failure (EASL-CLIF) reported that patients in the pre-ACLF phase of LC-AD who progressed to ACLF within 90 days had 3-month and 1-year mortality rates of 53.7% and 67.4%, respectively, which were mainly associated with a systemic inflammatory response [1, 2, 4]. Therefore, timely differentiation of the LC-AD population at high risk of developing ACLF in the short term facilitates early prevention and intervention, thus reducing mortality in patients with cirrhosis [5].

Various models have been used to predict disease progression in patients with chronic liver disease, including the Child–Turcotte–Pugh (CTP) model, model for end-stage liver disease (MELD), MELD-Na, and chronic liver failure consortium-acute decompensation score (CLIF-C ADs) [6–8]. However, these prediction models are usually based on partial serological indices and the subjective judgments of clinicians, and the cumbersome formulae and excessive parameters introduce additional confounding factors and limit clinical applications [9–11]. Therefore, the development of an objective, sensitive, and convenient index for predicting disease progression in patients with LC-AD is a challenge for current research. Previously, extracellular volume (ECV) calculated based on contrast-enhanced CT played a crucial role in assessing liver fibrosis and liver function [12, 13]. ECV represents the sum of extravascular and intravascular extracellular gaps. Progression of liver disease leads to an increase in hepatic collagen deposition, which enlarges the extracellular gap. Given this pathological basis, quantitative measurements of hepatic ECV can assess the severity of liver disease and predict disease progression [14, 15]. Advancements in dual-energy CT have enabled simultaneous evaluation of the liver parenchyma, hepatic blood flow, and liver function [16]. Recent studies have reported that ECV measured via dual-energy CT leads to improvements in staging liver fibrosis and predicting the development of decompensation and HCC in patients with compensated cirrhosis, when compared with conventional enhanced CT [17, 18].

Nomogram can incorporate a variety of prognostic-related factors and quantify and visualize the impact of each factor on prognosis and are more widely used in clinical practice. Hence, our study aimed to determine whether dual-energy CT measurements of ECV can be used to predict the occurrence of ACLF within 90 days in patients with HBV LC-AD and develop and validate a novel ECV-based prognostic model.

## Materials and methods

### Patients

**Fig. 1:**
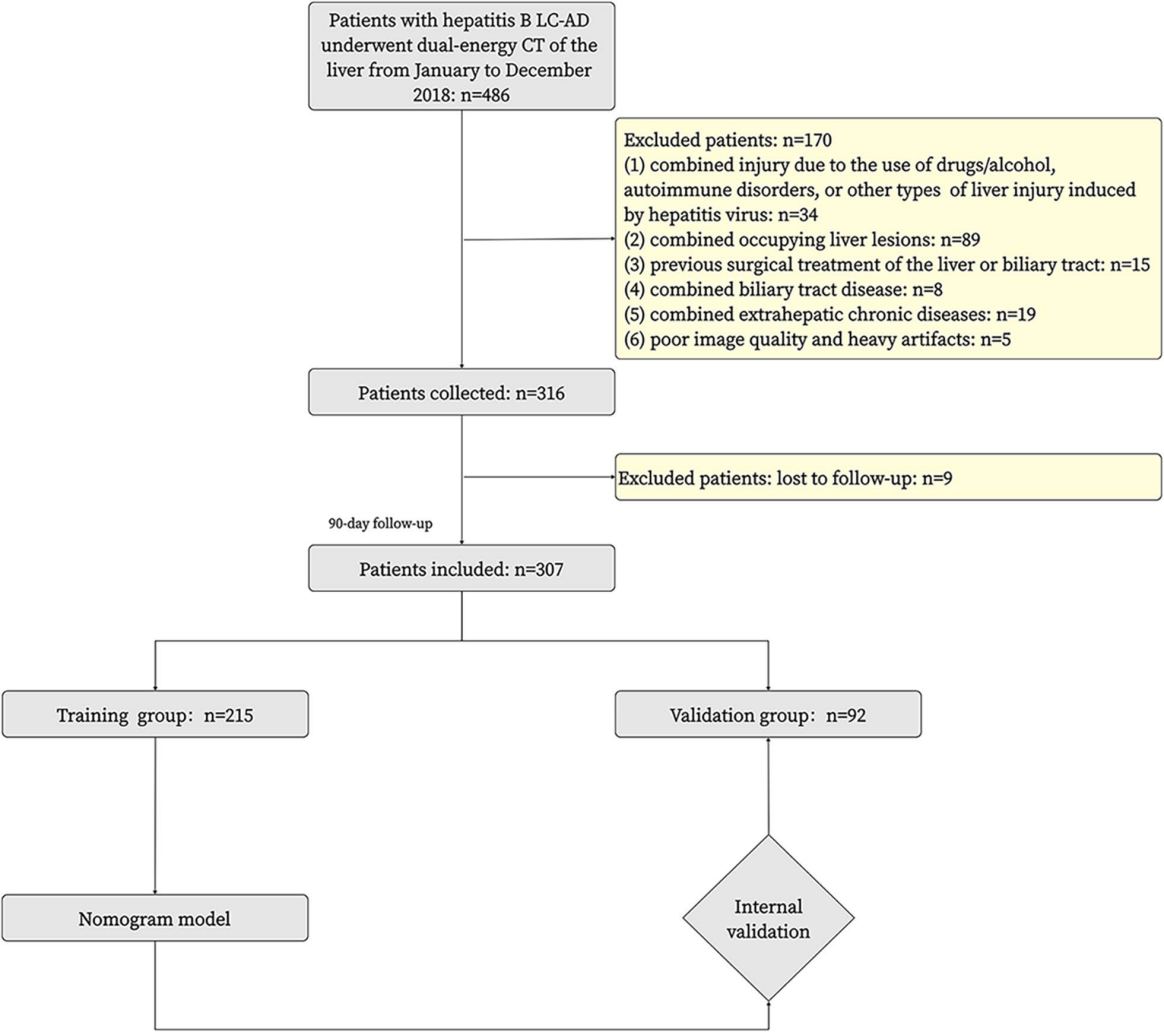
Flowchart of patient selection. LC-AD, liver cirrhosis-acute decompensation; ACLF, acute-on-chronic liver failure

This study was approved by our hospital ethics committee (No. 2021A-564), who waived the requirement written informed consent. We retrospectively collected data for 486 patients with HBV LC-AD hospitalized in our hepatology department between January 2018 and March 2022. Inclusion criteria were as follows: (1) clinical diagnosis of HBV LC-AD [19]; (2) age ≥ 18 years and ≤ 80 years; (3) availability of dual-energy enhanced CT scans of the liver; (4) complete laboratory test data within 1 week before and after the CT examination. Exclusion criteria were as follows: (1) combined injury due to the use of drugs/alcohol, autoimmune disorders, or other types of liver injury induced by hepatitis viruses; (2) combined occupying liver lesions (lesions in the liver with a single lesion or multiple lesions with a combined diameter of 3 cm, including hepatocellular carcinoma, cholangiocarcinoma, hepatic hemangioma, etc.); (3) previous surgical treatment of the liver or biliary tract; (4) combined biliary tract disease; (5) combined extrahepatic chronic diseases (e.g., malignant tumors in other organs; serious diseases affecting the heart, lung, kidney, brain, or other organs; uncontrolled immune or metabolic diseases); (6) poor image quality and heavy artifacts; (7) loss to follow-up. A final total of 307 patients with HBV LC-AD were included based on these inclusion and exclusion criteria, and patients were randomly assigned to the training and validation groups in a 7:3 ratio (Fig. 1).

### Data collection and follow-up

The clinical data of the patients are presented in Table 1, and the CTP, MELD, MELD-Na, and CLIF-C ADs were calculated based on the clinical data [8].

**Table 1:** Clinical characteristics, laboratory indices and equilibrium phase dual-energy CT parameters of patients in the training and validation groups

Variable	Total (*n* = 307)	Training Group (*n* = 215)	Validation Group (*n* = 92)	T/U/*χ*^2^ value	*p* value
Age (years)	50.1 ± 9.5	50.0 ± 9.8	50.4 ± 9.0	− 0.405	0.686
Sex (male/female)	195/112	138/77	57/35	0.138	0.710
BMI (kg/m^2^)	23.4 ± 3.2	23.3 ± 3.2	23.6 ± 3.3	− 0.851	0.395
Infections, *n* (%)	107 (34.9%)	72 (33.5%)	35 (38.0%)	0.589	0.443
Gastrointestinal bleeding, *n* (%)	79 (25.7%)	55 (25.6%)	24 (26.1%)	0.009	0.926
Ascites, *n* (%)	239 (77.9%)	167 (77.7%)	72 (78.3%)	0.013	0.910
Hepatic encephalopathy, *n* (%)	13 (4.2%)	8 (3.7%)	5 (5.4%)	0.467	0.495
History of HBV (years)	2.0 (0.0, 10.7)	2.7 (0.0, 10.8)	0.8 (0.0, 10.6)	− 1.205	0.228
Antiviral therapy, *n* (%)	117 (38.1%)	88 (40.9%)	29 (31.5%)	2.418	0.120
Antiviral during follow-up, *n* (%)	217 (70.7%)	155 (72.1%)	62 (67.4%)	0.687	0.407
HBeAg, *n* (%)					
Positive	204 (66.4%)	134 (62.3%)	70 (76.1%)	5.473	0.019
Negative	103 (33.6%)	81 (37.7%)	22 (23.9%)		
Lg HBV-DNA (copies/mL)	4.0 (2.5, 6.1)	3.5 (2.5, 6.0)	4.9 (2.7, 6.3)	− 1.908	0.056
Urea (mmol/L)	5.4 (4.3, 7.0)	5.5 (4.3, 7.0)	5.4 (4.1, 7.2)	− 0.434	0.664
Creatinine (umol/L)	66.0 (57.0, 77.0)	66.0 (57.0, 77.0)	67.0 (56.1, 78.3)	− 0.178	0.858
Na^+^ (mmol/L)	139.0 (137.0, 141.0)	139.0 (137.0, 141.0)	139.0 (137.0, 141.0)	− 0.058	0.954
ALT (U/L)	35.0 (24.0, 52.0)	35.0 (24.0, 50.0)	35.0 (25.3, 52.8)	− 0.376	0.707
AST (U/L)	45.0 (31.0, 65.0)	44.0 (30.0, 65.0)	45.5 (32.3, 65.8)	− 0.514	0.607
GGT (U/L)	33.0 (20.0, 61.0)	32.0 (20.0, 61.0)	35.5 (21.3, 61.8)	− 0.622	0.534
ALP (U/L)	103.0 (82.0, 130.0)	102.0 (82.0, 130.0)	111.0 (86.0, 132.3)	− 0.926	0.355
ALB (g/L)	33.1 ± 6.2	33.6 ± 6.2	31.8 ± 6.2	2.275	0.024
TB (umol/L)	28.5 (17.8, 42.3)	28.5 (18.0, 43.3)	29.5 (17.7, 42.1)	− 0.156	0.876
Cholesterol (mmol/L)	2.6 (2.1, 3.1)	2.6 (2.1, 3.1)	2.6 (2.1, 3.2)	− 0.038	0.970
WBC (10^9/L)	3.6 (2.3, 4.8)	3.4 (2.3, 4.8)	3.7 (2.3, 4.8)	− 0.482	0.630
PLT (10^9/L)	56.0 (39.0, 74.0)	54.0 (37.0, 73.0)	59.0 (42.3, 82.5)	− 1.545	0.122
HCT (L/L)	0.35 ± 0.08	0.35 ± 0.08	0.34 ± 0.07	0.657	0.512
PT (s)	14.9 (13.4, 16.4)	14.8 (13.4, 16.3)	15.0 (13.3, 16.4)	− 0.263	0.793
PTA (%)	62.6 (53.8, 74.2)	62.6 (53.8, 74.2)	63.1 (54.1, 75.1)	− 0.135	0.892
INR	1.3 (1.2, 1.4)	1.3 (1.2, 1.4)	1.3 (1.2, 1.4)	− 0.828	0.408
CTP score	8.0 (6.0, 9.0)	8.0 (6.0, 9.0)	8.0 (7.0, 9.0)	− 1.498	0.134
MELD score	11.5 (9.1, 13.9)	11.4 (9.1, 13.9)	11.5 (9.1, 14.4)	− 0.466	0.641
MELD-Na score	12.2 (10.1, 15.5)	12.2 (10.1, 15.5)	12.1 (10.0, 16.4)	− 0.214	0.831
CLIF-C ADs	38.6 (34.2, 44.3)	38.6 (33.8, 44.5)	38.6 (34.7, 43.5)	− 0.108	0.914
IC	20.3 ± 4.5	20.1 ± 4.6	20.7 ± 4.3	− 1.176	0.240
Z	8.8 ± 0.2	8.7 ± 0.2	8.8 ± 0.2	− 1.194	0.234
K_140_	1.6 ± 0.4	1.6 ± 0.4	1.6 ± 0.3	− 1.113	0.267
ECV_IC-liver_	33.1 ± 5.5	33.0 ± 5.8	33.5 ± 5.0	− 0.725	0.469
90-day ACLF development, *n* (%)	65 (21.2%)	47 (21.9%)	18 (19.6%)	0.203	0.652

*BMI* body mass index, *ALT* alanine aminotransferase, *AST* aspartate aminotransferase, *GGT* gamma-glutamyl transpeptidase, *ALP* alkaline phosphatase, *ALB* albumin, *TB* total bilirubin, *WBC* white blood cells, *PLT* platelet count, *HCT* hematocrit, *PT* prothrombin time, *PTA* prothrombin activity, *INR* international normalized ratio, *MELD* model of end-stage liver disease, *CLIF-C ADs* chronic liver failure consortium-acute decompensation score, *IC* iodine concentration, *Z* effective atomic number, *K*_*140*_ slope of the energy spectrum curve, *ECV*_*IC-liver*_ extracellular liver volume, *ACLF* acute-on-chronic liver failure

All patients were given conventional comprehensive internal medicine treatment (such as rest, liver protection, infusion of albumin or plasma, maintenance of water and electrolyte balance, and nutritional and energy support treatment), and/or antiviral therapy (e.g., lamivudine, telbivudine, or entecavir) after admission. The follow-up period for this study was 90 days, and the follow-up was performed through the electronic medical record system. The progressive group included patients who required readmission for ACLF within 90 days after diagnosis of LC-AD, while the stable group included patients who did not (Fig. 1). ACLF was diagnosed according to the consensus criteria of the Asian-Pacific Association for the Study of the Liver (APASL): Jaundice (serum bilirubin ≥ 5 mg/dL) and coagulation disorders (international normalized ratio ≥ 1.5 or prothrombin activity < 40%) complicated by ascites and/or encephalopathy within 4 weeks [4].

### Image acquisition

All patients underwent standardized liver scans using a 64-row dual-energy CT (Discovery CT750 HD, GE Healthcare, Waukesha, WI, USA). Gemstone Spectral Imaging (GSI) Scan Mode with a fast switching of tube voltage 80/140 kVp (switching rate: 0.5 ms), tube current: 375 mA, spiral speed: 0.6 s/r, acquisition thickness 5 mm, acquisition intervals 5 mm, pitch 0.969: 1, and detector collimation: 0.625 × 64 mm. A high-pressure syringe was used to inject non-ionic contrast medium (Ultravist 370; Bayer Schering Pharma, Berlin, Germany) through the anterior elbow vein at a dose of 60–90 mL (1.0 mL/kg body weight), and injection flow rate: 3.5–4.0 mL/s. The abdominal aorta was detected at a trigger threshold of 100 HU and scanned at 20 s, 60 s and 180 s after triggering the pulse phase, portal phase, and equilibrium phase. At the end of the scan, images from each phase were reconstructed with the thickness and intervals of 1.25 mm.

### Image analysis

All data were transferred to a workstation (GE AW4.7, GE Healthcare, Waukesha, WI, USA) and processed for analysis using GSI Volume Viewer software. Regions of interest (ROIs) were drawn on cross-sectional iodine (water) images of the liver at equilibrium by two physicians (with more than 10 years of experience in diagnostic liver imaging).

Circular ROIs (diameter 10 mm) were drawn in the left outer lobe, left inner lobe, right anterior lobe, right posterior lobe of the liver and in the abdominal aorta at the same level, centered on the first hepatic hilar at three different levels (at the level of the hilar and three levels 5 mm above and below it). The ROI should be outlined to avoid major vessels, bile ducts, cysts and artifacts as much as possible. Single-energy CT values at 40 keV ~ 140 keV, iodine concentration (IC), and effective atomic number (Z) were measured and recorded in different liver lobes and abdominal aorta. The data were used to calculate the slope of the energy spectrum curve (K_140_) [20] and extracellular liver volume (ECV_IC-liver_) [21]. The average values of the four liver ROIs were used for liver parenchyma measurements, and values for each site in three different levels were averaged.

$${\text{K}}_{140} = \frac{{{\text{CTvalue}}_{{40\;{\text{kev}}}} - {\text{CTvalue}}_{{140{\text{kev}}}} }}{100}$$

$${\text{ECV}}_{{\text{IC - liver}}} = \frac{{{\text{IC}}_{{{\text{liver}}}} \times \left( {100 - {\text{HCT}}\% } \right)}}{{{\text{IC}}_{{{\text{aorta}}}} }}$$

### Statistical analysis

IBM SPSS (version 25; IBM, New York, USA) and R software (version 4.2.1) were used for statistical analysis. The agreement between the measured parameters of the two physicians was evaluated using intraclass correlation coefficients (ICCs), and ICCs values ≥ 0.75 were considered good. The Kolmogorov–Smirnov normality test was performed, and data conforming to a normal distribution were expressed as the mean ± standard deviation ($\overline{\chi } \pm s$) and compared between groups using the independent samples t-test; those that did not conform to a normal distribution were expressed as the median and interquartile range (Q1, Q3) and compared between groups using the Mann–Whitney U test. Count data were expressed as frequencies and percentages *n* (%), and the *χ*^2^ test was used for comparison.

Variables that significantly differed (*p* < 0.05) between the progressive and stable groups in the univariate analysis were entered into a multivariate logistic regression model using a enter method to identify independent risk factors predicting progression and to create a model to predict disease progression. Multicollinearity among variables was measured according to the variance inflation factor (VIF), and this study considered a high risk of multicollinearity at a VIF of ≥ 10. A validation group was used to internally validate the model, and the receiver operating characteristic (ROC) curves and the area under the curve (AUC) to evaluate the discrimination of the model on the outcome variable. Model calibration was assessed using the Hosmer–Lemeshow (H–L) test and calibration curve, and the decision analysis curve (DCA) was used to assess the clinical utility and net benefit of the model. *p* values < 0.05 were considered to indicate a statistically significant difference.

## Results

### Patient characteristics

307 patients with HBV LC-AD were included in our study. There were 215 cases in the training group and 92 cases in the validation group. The clinical characteristics, laboratory indices and equilibrium phase dual-energy CT parameters of the patients in the training and validation groups are shown in Table 1. The differences in ALB and HBeAg-positive between the two groups were statistically significant (*p* < 0.05), while differences between groups for the remaining indicators were not statistically significant (*p* > 0.05).

### Univariable and multivariable predictors of ACLF development

**Fig. 2:**
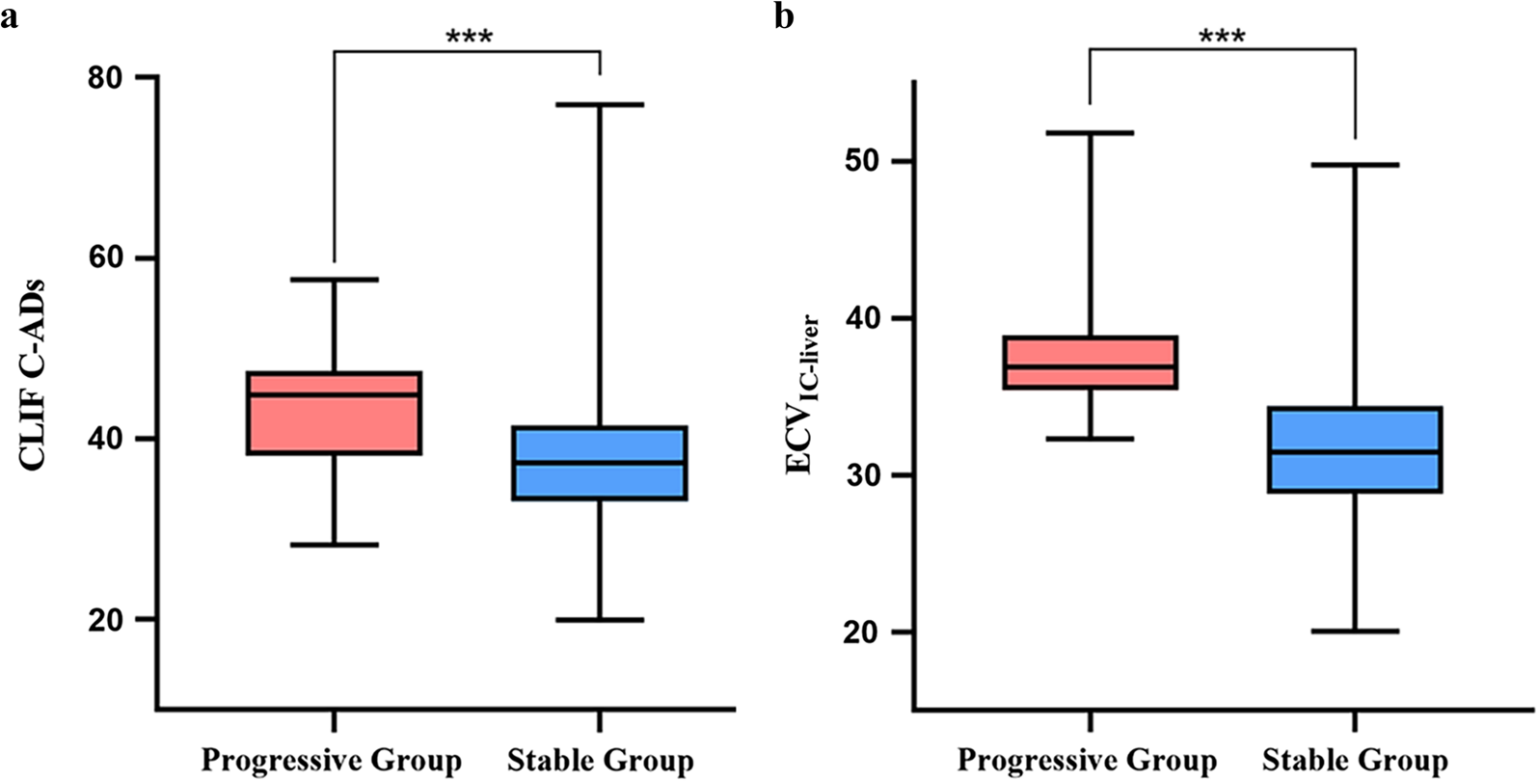
Box plots of CLIF-C ADs and ECV_IC-liver_ for the progressive and stable groups. CLIF-C ADs, chronic liver failure consortium-acute decompensation score; ECV_IC-liver_, extracellular liver volume; ***, *p* < 0.001

In the training group, univariate analysis showed that age, BMI, infection, ascites, urea, Na^+^, ALB, WBC, PLT, CTP score, MELD-Na score, CLIF-C ADs, IC, Z, K_140_, and ECV_IC-liver_ were risk factors associated with the development of ACLF within 90 days in patients with HBV LC-AD (*p* < 0.05, Table 2). In the multivariable logistic regression analysis, MELD score, MELD-Na score, IC, Z, and K_140_ were excluded because of their strong risk of covariance (VIF values of 52.30, 65.89, 159.45, 414.02, and 459.46, respectively). Among the remaining indicators, CLIF-C ADs (OR 1.188; 95% CI 1.046–1.350;* p* = 0.008) and ECV_IC- liver_ (OR 1.280; 95% CI 1.154–1.420; *p* < 0.001) were identified as independent predictors (Table 3 and Fig. 2). Representative cases of the progressive and stable groups are shown in Additional file 1: Figs. S1 and S2.

**Table 2:** Univariate analysis of risk factors associated with the occurrence of ACLF within 90 days

Variable	Training Group (*n* = 215)			Validation Group (*n* = 92)		
	Progressive Group (*n* = 47)	Stable Group (*n* = 168)	*p* value	Progressive Group (*n* = 18)	Stable Group (*n* = 74)	*p* value
Age (years)	52.7 ± 11.2	49.2 ± 9.2	0.031	53.4 ± 9.9	49.7 ± 8.7	0.114
Sex (male/female)	33/14	105/63	0.330	11/7	46/28	0.934
BMI (kg/m^2^)	22.5 ± 3.0	23.5 ± 3.2	0.051	22.1 ± 2.5	24.0 ± 3.4	0.031
Infections, *n* (%)	25 (53.2%)	47 (28.0%)	0.001	12 (66.7%)	23 (31.1%)	0.005
Gastrointestinal bleeding, *n* (%)	15 (31.9%)	40 (23.8%)	0.260	5 (27.8%)	19 (25.7%)	0.855
Ascites, *n* (%)	45 (95.7%)	122 (72.6%)	0.001	18 (100.0%)	54 (73.0%)	0.013
Hepatic encephalopathy, *n* (%)	2 (4.3%)	6 (3.6%)	0.827	1 (5.6%)	4 (5.4%)	1.000
History of HBV (years)	1.0 (0.0, 10.0)	3.0 (0.1, 10.8)	0.192	0.8 (0.0, 11.5)	1.3 (0.0, 10.6)	0.948
Antiviral therapy, *n* (%)	14 (29.8%)	74 (44.0%)	0.079	4 (22.2%)	25 (33.8%)	0.507
Antiviral during follow-up, *n* (%)	36 (76.6%)	119 (70.8%)	0.436	12 (66.7%)	50 (67.6%)	0.942
HBeAg-positive, *n* (%)	33 (70.2%)	101 (60.1%)	0.207	17 (94.4%)	53 (71.6%)	0.084
Lg HBV-DNA (copies/mL)	4.0 (2.5,6.4)	3.2 (2.5,5.7)	0.102	5.5 (3.4,7.0)	4.8 (2.6, 5.9)	0.067
Urea (mmol/L)	6.7 (4.8, 9.1)	5.3 (4.3, 6.6)	0.001	7.2 (4.3, 8.9)	5.2 (4.1, 6.5)	0.086
Creatinine (umol/L)	70.0 (59.0, 80.4)	64.5 (57.0, 75.0)	0.194	70.0 (59.8, 87.3)	63.5 (56.0, 75.3)	0.174
Na^+^ (mmol/L)	137.4 (135.6, 139.5)	139.0 (137.0, 141.0)	0.001	138.0 (135.7, 141.0)	139.0 (137.0,141.0)	0.300
ALT (U/L)	36.0 (24.0, 58.0)	34.0 (24.0, 48.0)	0.265	34.0 (27.5, 59.5)	35.0 (24.8, 53.0)	0.451
AST (U/L)	45.0 (29.0, 86.0)	44.0 (31.0, 63.8)	0.488	51.5 (35.8, 78.5)	42.5 (31.0, 64.3)	0.304
GGT (U/L)	29.0 (19.0, 92.0)	32.0 (20.3, 54.0)	0.560	31.5 (19.8, 98.3)	36.0 (21.8, 61.0)	0.949
ALP (U/L)	100.0 (77.0, 131.0)	102.5 (82.0, 127.8)	0.617	97.5 (80.8, 125.5)	112.0 (86.8, 134.0)	0.395
ALB (g/L)	31.0 ± 5.7	34.3 ± 6.2	0.001	28.5 ± 5.0	32.6 ± 6.2	0.010
TB (umol/L)	30.2 (19.9, 50.6)	26.8 (17.4, 41.0)	0.138	34.4 (24.4, 49.2)	26.3 (17.3, 41.5)	0.097
Cholesterol (mmol/L)	2.5 (2.0, 2.9)	2.7 (2.2, 3.2)	0.100	2.4 (2.0, 2.9)	2.7 (2.1, 3.3)	0.115
WBC (10^9/L)	4.7 (3.4, 7.6)	3.2 (2.1, 4.3)	< 0.001	4.5 (3.7, 6.0)	3.4 (2.2, 4.5)	0.008
PLT (10^9/L)	66.0 (54.0, 86.0)	49.5 (34.0, 71.0)	0.001	63.0 (54.8, 73.0)	58.0 (41.5, 85.8)	0.445
HCT (L/L)	0.34 ± 0.08	0.35 ± 0.08	0.245	0.34 ± 0.08	0.35 ± 0.07	0.717
PT (s)	15.2 (13.7, 15.5)	14.6 (13.3, 16.4)	0.390	15.3 (14.8, 17.4)	14.7 (13.3, 16.3)	0.089
PTA (%)	64.1 (55.3, 70.0)	62.2 (52.5, 78.0)	0.861	57.5 (49.5, 67.8)	64.2 (55.7, 78.5)	0.174
INR	1.3 (1.2, 1.4)	1.3 (1.2, 1.4)	0.278	1.3 (1.3, 1.5)	1.3 (1.2, 1.4)	0.137
CTP score	9.0 (7.0, 9.0)	7.0 (6.0, 9.0)	< 0.001	9.0 (7.8, 10.3)	8.0 (6.0, 9.0)	0.009
MELD score	12.1 (10.1, 16.8)	11.2 (8.8, 13.7)	0.063	13.0 (9.9, 17.1)	11.5 (8.9, 13.9)	0.086
MELD-Na score	14.1 (10.8, 18.9)	11.9 (10.0, 14.7)	0.011	14.3 (10.0, 19.9)	12.0 (10.0, 15.6)	0.184
CLIF-C ADs	44.8 (38.1, 47.5)	37.3 (33.0, 41.5)	< 0.001	41.8 (37.8, 44.7)	38.5 (34.2, 42.6)	0.019
IC	22.6 ± 5.0	19.4 ± 4.2	< 0.001	23.2 ± 5.3	20.1 ± 3.9	0.007
Z	8.9 ± 0.3	8.7 ± 0.2	< 0.001	8.9 ± 0.3	8.7 ± 0.2	0.008
K_140_	1.7 ± 0.4	1.5 ± 0.3	< 0.001	1.8 ± 0.4	1.6 ± 0.3	0.008
ECV_IC-liver_	38.2 ± 4.8	31.5 ± 5.1	< 0.001	37.3 ± 3.1	32.6 ± 4.9	< 0.001

*BMI* body mass index, *ALT* alanine aminotransferase, *AST* aspartate aminotransferase, *GGT* gamma-glutamyl transpeptidase, *ALP* alkaline phosphatase, *ALB* albumin, *TB* total bilirubin, *WBC* white blood cells, *PLT* platelet count, *HCT* hematocrit, *PT* prothrombin time, *PTA* prothrombin activity, *INR* international normalized ratio, *MELD* model of end-stage liver disease, *CLIF-C ADs* chronic liver failure consortium-acute decompensation score, *IC* iodine concentration, *Z* effective atomic number, *K*_*140*_ slope of the energy spectrum curve, *ECV*_*IC-liver*_ extracellular liver volume, *ACLF* acute-on-chronic liver failure

**Table 3:** Multivariate logistic regression analysis of risk factors associated with the occurrence of ACLF within 90 days based on the training group

Variable	β	BE	Walds	*p* value	OR	95% CI
Age (years)	0.007	0.026	0.064	0.801	1.007	0.956–1.059
BMI (kg/m^2^)	− 0.105	0.073	2.074	0.150	0.900	0.781–1.039
Infections, *n* (%)	0.708	0.477	2.201	0.138	2.030	0.797–5.175
Ascites, *n* (%)	2.090	1.128	3.432	0.064	8.087	0.886–73.842
Urea (mmol/L)	0.044	0.038	1.357	0.244	1.045	0.970–1.126
Na^+^ (mmol/L)	− 0.025	0.069	0.136	0.713	0.975	0.852–1.115
ALB (g/L)	0.023	0.053	0.186	0.666	1.023	0.923–1.134
WBC (10^9/L)	− 0.039	0.063	0.375	0.540	0.962	0.850–1.089
PLT (10^9/L)	0.003	0.005	0.313	0.576	1.003	0.993–1.012
CTP score	− 0.191	0.225	0.716	0.398	0.826	0.531–1.285
CLIF-C ADs	0.172	0.065	6.999	0.008	1.188	1.046–1.350
ECV_IC-liver_	0.247	0.053	21.874	< 0.001	1.280	1.154–1.420
Constant	− 13.035	10.365	1.582	0.209	0.000	

*ACLF* acute-on-chronic liver failure, *BMI* body mass index, *ALB* albumin, *WBC* white blood cells, *PLT* platelet count, *MELD* model of end-stage liver disease, *CLIF-C ADs* chronic liver failure consortium-acute decompensation score, *ECV*_*IC-liver*_ extracellular liver volume, *OR* odds ratio, *CI* confidence interval

### Development of the nomogram

**Fig. 3:**
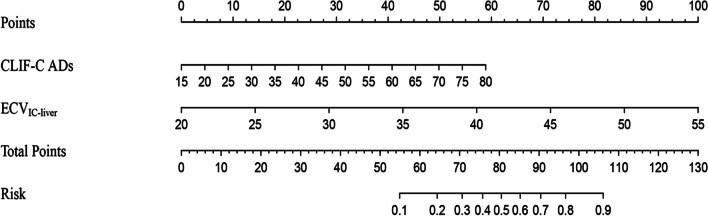
The nomogram for predicting the occurrence of ACLF within 90 days. CLIF-C ADs, chronic liver failure consortium-acute decompensation score; ECV_IC-liver,_ extracellular liver volume; ACLF, acute-on-chronic liver failure

Two predictors (CLIF C-ADs and ECV_IC-liver_) were screened based on multivariate logistic regression analysis, and a nomogram of predicting the occurrence of ACLF within 90 days in HBV LC-AD patients was established (Table 3, Fig. 3). Each variable is assigned a specific score, and the sum of the scores of all variables corresponds to the probability of ACLF risk.

### Validation of the nomogram

**Fig. 4:**
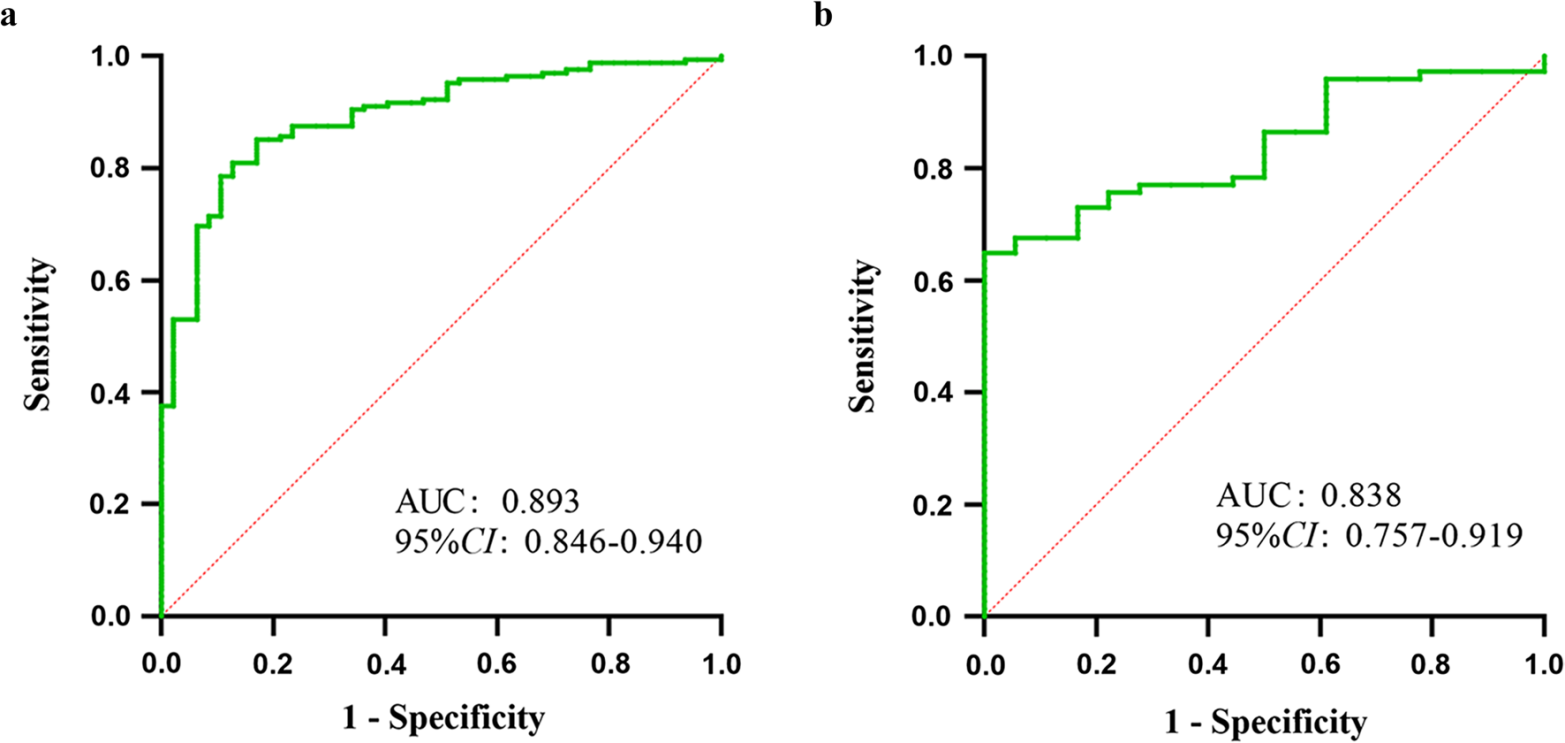
The ROC curves for predicting the occurrence of ACLF within 90 days. The AUC value of model was 0.893 (95% CI 0.846–0.940) in the training group (**a**) and 0.838 (95% CI 0.757–0.919) in the validation group (**b**). ROC, receiver operating characteristic; ACLF, acute-on-chronic liver failure; AUC, area under the curve

**Fig. 5:**
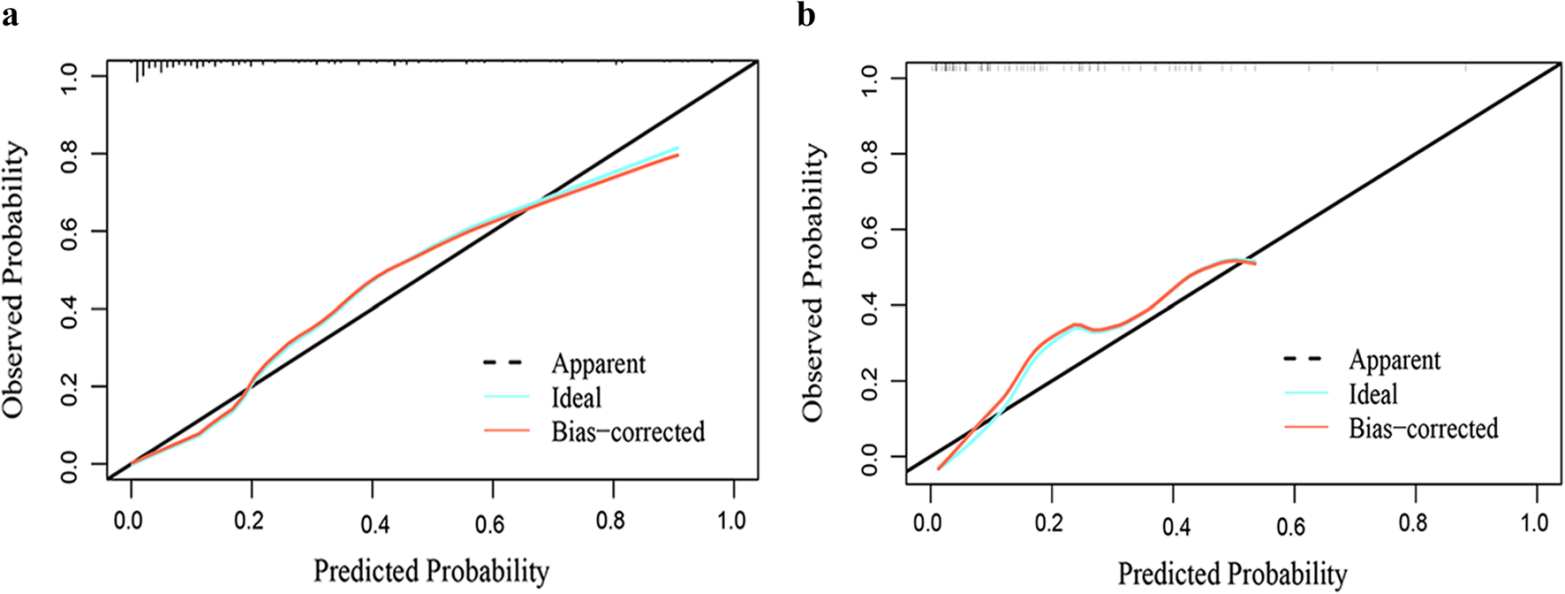
The calibration curve for predicting the occurrence of ACLF within 90 days. The calibration curves of the model in the training group (**a**) and validation group (**b**) showed a good consistency. ACLF, acute-on-chronic liver failure

**Fig. 6:**
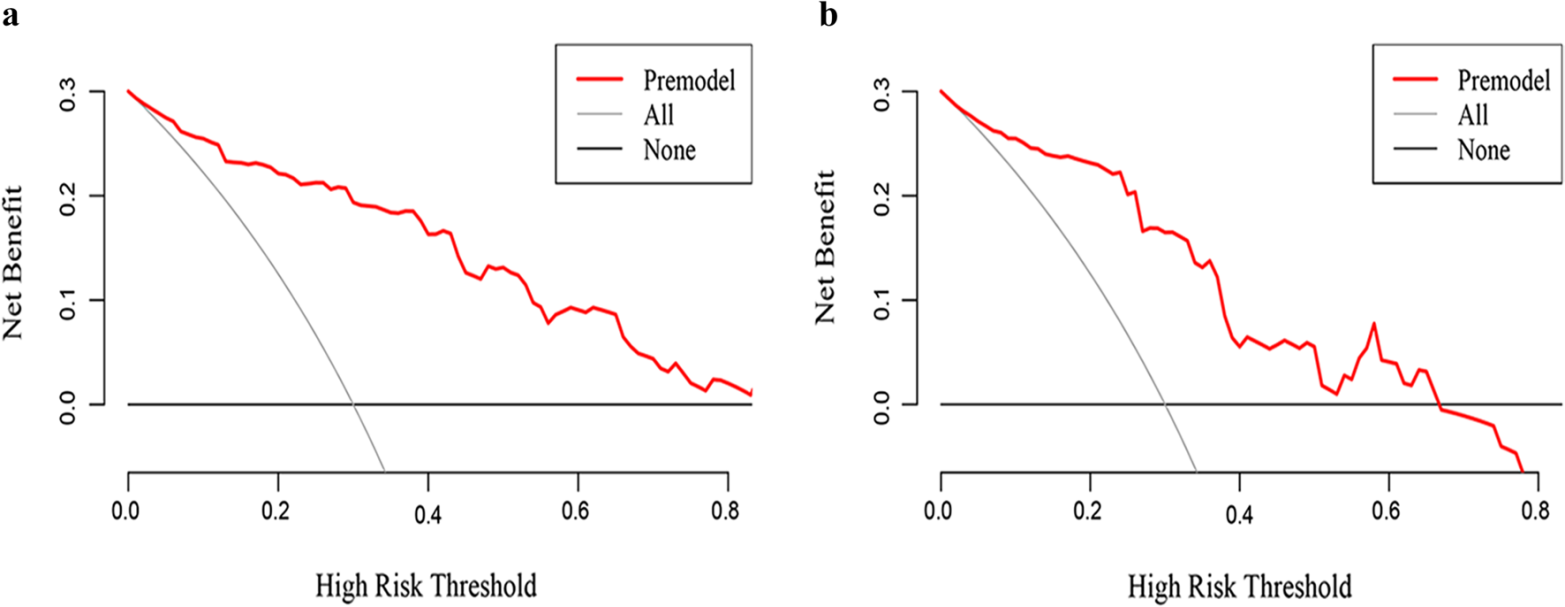
The DCA curve for predicting the occurrence of ACLF within 90 days. DCA analysis showed that the model had an overall good net benefit within the wide and practical ranges of threshold probabilities and influenced patient outcomes. DCA, decision analysis curve; ACLF, acute-on-chronic liver failure

Internal validation of the nomogram by the validation group showed that the model had an AUC value of 0.893 (95% CI 0.846–0.940) in the training group and 0.838 (95% CI 0.757–0.919) in the validation group, indicating that the model has good discrimination in predicting the occurrence of ACLF within 90 days in HBV LC-AD patients (Fig. 4). The calibration curves were very close to the diagonal line, indicating a good agreement between the predicted probability of model and the actual results; moreover, the H–L tests all obtained insignificant *p*-values of 0.658 and 0.366 for the training and validation groups, respectively (Fig. 5). The DCA results showed a higher net benefit for the model over almost the entire range of threshold probabilities compared with both the variable-all and variable-none scenarios, indicating the higher clinical utility of the model (Fig. 6).

## Discussion

In clinical practice, predicting the occurrence of ACLF within 90 days in patients with LC-AD is extremely challenging [2]. In this study, ECV_IC-liver_, which was determined using iodine (water) maps obtained in the equilibrium phase of dual-energy CT, and CLIF-C ADs were identified as an independent risk factor for the progression of LC-AD in the univariate and multivariable logistic regression analysis, and the model constructed by the combination of the two could be used for predicting the occurrence of ACLF within 90 days in patients with HBV LC-AD. The performance of model achieved better results in both the training and validation groups, which may help to provide a more appropriate and intuitive assessment tool for the clinical management of LC-AD patients.

The recent study reported that patients with LC-AD who developed ACLF within 90 days had more severe systemic inflammation than patients with stable disease, with a short-term mortality rate of 53.7% [1, 2]. Several previous studies have successfully predicted prognosis in patients with cirrhosis by monitoring liver function, inflammatory status, and portal hypertension [22–24]. Therefore, it is theoretically possible to predict the occurrence of ACLF in patients with LC-AD based on pathophysiological status. In this study, significant differences in several clinical indicators (infection, ascites, urea, Na^+^, ALB, WBC, PLT, etc.) were observed between the progressive and stable groups, which may to some extent reflect changes in systemic inflammation and portal pressure, as suggested in previous studies [25, 26]. However, the use of serum markers and clinical symptoms to assess the prognosis of cirrhosis still has certain limitations (e.g., susceptibility to extrahepatic factors, subjectivity, and instability of evaluation indices), making these data unsuitable for predicting prognosis in patients with LC-AD [9–11]. Therefore, we evaluated the development of 90-day ACLF in these patients using the newly proposed CLIF-C ADs, which was also independently associated with patient prognosis. In contrast to conventional CTP, MELD and MELD-Na scores, the CLIF-C ADs was proposed based on LC-AD specifically and takes into account the value of WBC and age for prognostic evaluation [8]. WBC reflects systemic inflammatory status, while age is negatively correlated with total body muscle mass, so a novel score combining these two factors can help to determine prognostic risk in patients with LC-AD [27].

ECV is a marker used to quantify the status of the extracellular matrix, which is an important component of the cellular microenvironment, and the development of disease is usually accompanied by changes in the cellular microenvironment. Therefore, ECV can reflect the extent of tissue inflammation, liver function, and the degree of fibrosis [21, 28, 29]. In the dynamic progression of cirrhosis, the gradual deposition of collagen in the liver leads to gradual expansion of the extracellular gap. After the distribution of the imaging agent in and out of cells and blood vessels during the equilibrium phase reaches stability, ECV can be quantified by evaluating the distribution of the imaging agent during the equilibrium phase [30]. Several studies initially used conventional contrast-enhanced CT to measure ECV, reporting that liver and spleen ECV values allow for non-invasive assessment of the extent of organ fibrosis and the severity of portal hypertension, but with low diagnostic efficacy [13, 31]. Recently, some studies have further used dual-energy CT equilibrium phase iodine maps to measure ECV, which significantly improves the accuracy of extracellular matrix measurements based on dual-energy CT iodine maps when compared with conventional CT values, making them effective tools for the noninvasive assessment of organ fibrosis and prediction of disease progression [17, 32]. In addition, most previous studies [21, 30, 31, 33] have focused on the use of ECV for staging liver fibrosis, grading the severity of portal hypertension, and predicting the occurrence of decompensation and HCC, while none have attempted to predict the occurrence of ACLF within 90 days in patients with LC-AD. Therefore, we used ECV_IC-liver_ values quantified using dual-energy CT to predict short-term disease progression in patients with LC-AD. Our analysis indicated that ECV_IC-liver_ was independently associated with the occurrence of ACLF at 90 days, and that patients with worse prognosis had higher ECV_IC-liver_ values than patients moving toward stable disease. Our model also demonstrated excellent predictive power. In addition, conventional dual-energy CT parameters (IC, Z, K_140_, etc.) can be obtained simultaneously when measuring ECV_IC-liver_, enabling the assessment of hepatic blood flow and liver function. However, the above parameters measured in this study were excluded because of the risk of covariance, probably due to the limited sample size in this single-center population and the fact that the study only measured single-phase dual-energy CT parameters of patients, which reflect relatively limited information about the liver, and multiphase dual-energy CT parameters of patients from multiple centers could be included in the future to reflect the liver status more comprehensively.

In this study, ECV_IC-liver_ and CLIF-C ADs increased with disease progression in patients with LC-AD and differed significantly between the progressive and stable groups. Recent studies have reported that the diagnostic efficacy of ECV for predicting prognosis in patients with cirrhosis is comparable or slightly better than that of CTP and MELD scores and that dynamic monitoring of hepatic ECV is beneficial for patient prognosis [33]. Bak et al. [18] demonstrated that hepatic ECV values increased linearly with increasing cirrhosis stage using dual-energy CT, and its sensitivity and accuracy for the non-invasive prediction of liver decompensation and HCC were higher than those for the MELD score. This finding suggests that dual-energy CT is a reliable and sensitive method for assessment and individualized management in patients with compensated cirrhosis. Baldin et al. [27] demonstrated that high CLIF-C ADs are associated with a higher incidence of organ dysfunction, increased risk of complications, and high short-term mortality, allowing for more accurate short-term prognostic prediction. In this study, we further combined the CLIF-C ADs, which reflects the clinical index, and the ECV_IC-liver,_ which reflects the state of the cellular microenvironment, to construct and validate a model for the occurrence of ACLF in LC-AD patients within 90 days. The AUC value of the model in the validation group was 0.838 and a good calibration curve was obtained, indicating that the model has good predictive value, and the satisfactory DCA curve indicates that the model has high clinical value, but future validation with larger sample sizes from multiple centers is still needed.

This study had several limitations. First, the CT scan protocol used in this study was limited to our center, and the results of ECV measured using different delay times may differ. The accuracy of ECV quantified at different delay times with respect to the extracellular matrix should be further explored in prospective studies. Second, this study included patients with HBV cirrhosis only, and the study follow-up period was only 90 days. Further expansion of the study population and extension of the follow-up period are required to identify additional imaging parameters that may reflect liver prognosis, such as liver and spleen volume, liver surface nodule score, paravertebral muscle density, and fat content. Finally, as a retrospective single-center study, the study may be biased due to the limited total sample size, and without external validation, the generalizability of the model requires more in-depth future studies.

In conclusion, the dual-energy CT quantitative ECV_IC-liver_ can achieve the prediction of ACLF occurrence within 90 days in patients with LC-AD; internal validation showed that the model constructed by combining ECV_IC-liver_ and CLIF-C ADs has good predictive performance and clinical utility. Therefore, the results of this study can be used to assist the clinic to better identify the early disease progression in LC-AD patients.

## Supplementary Information

**Additional file 1: Fig. S1.** (Progressive group) Dual-energy CT equilibrium phase imaging of the liver in a 58-year-old woman with HBV LC-AD. A-E respectively show the iodine (water) map, iodine (water) pseudo-color map, effective atomic number map, histogram and slope of the energy spectrum curve, with iodine concentration (IC) =22.84, effective atomic number (Z) =8.94, energy spectrum curve (K_140_) =1.76 and extracellular liver volume (ECV_IC-liver_) =36.74. **Fig. S2.** (Stable group) Dual-energy CT equilibrium phase imaging of the liver in a 34-year-old man with HBV LC-AD. A-E respectively show the iodine (water) map, iodine (water) pseudo-color map, effective atomic number map, histogram and slope of the energy spectrum curve, with iodine concentration (IC) =15.63, effective atomic number (Z) =8.52, energy spectrum curve (K_140_) =1.21 and extracellular liver volume (ECV_IC-liver)_ =26.13.

## Data Availability

The datasets used and/or analyzed during the current study are available from the corresponding author on reasonable request.

## Abbreviations

ACLF: Acute-on-chronic liver failure
AUC: Area under the curve
CLIF-C ADs: Chronic liver failure consortium-acute decompensation score
CTP: Child–Turcotte––Pugh
ECV_IC-liver_: Extracellular liver volume
HBV: Hepatitis B
VIF: Variance inflation factor
H–L: Hosmer–Lemeshow
DCA: Decision analysis curve
IC: Iodine concentration
K_140_: Energy spectrum curve slope
LC-AD: Liver cirrhosis-acute decompensation
MELD: Model for end-stage liver disease
ROC: Receiver operating characteristic
Z: Effective atomic number
