# Supplementary material for: Dual-energy CT quantification of extracellular liver volume predicts short-term disease progression in patients with hepatitis B liver cirrhosis-acute decompensation
Source: Insights Imaging. 2023 Mar 29;14:51. doi: 10.1186/s13244-023-01393-x (PMC10050608; doi:10.1186/s13244-023-01393-x)
Supplement: Supplementary file 1 — Additional file 1: Fig. S1. (Progressive group) Dual-energy CT equilibrium phase imaging of the liver in a 58-year-old woman with HBV LC-AD. A-E respectively show the iodine (water) map, iodine (water) pseudo-color map, effective atomic number map, histogram and slope of the energy spectrum curve, with iodine concentration (IC) =22.84, effective atomic number (Z) =8.94, energy spectrum curve (K140) =1.76 and extracellular liver volume (ECVIC-liver) =36.74. Fig. S2. (Stable group) Dual-energy CT equilibrium phase imaging of the liver in a 34-year-old man with HBV LC-AD. A-E respectively show the iodine (water) map, iodine (water) pseudo-color map, effective atomic number map, histogram and slope of the energy spectrum curve, with iodine concentration (IC) =15.63, effective atomic number (Z) =8.52, energy spectrum curve (K140) =1.21 and extracellular liver volume (ECVIC-liver) =26.13. [file 13244_2023_1393_MOESM1_ESM.pdf]

ELECTRONIC SUPPLEMENTARY MATERIAL

Dual-energy CT quantification of extracellular liver volume predicts short-term disease progression in patients with hepatitis B liver cirrhosis-acute decompensation

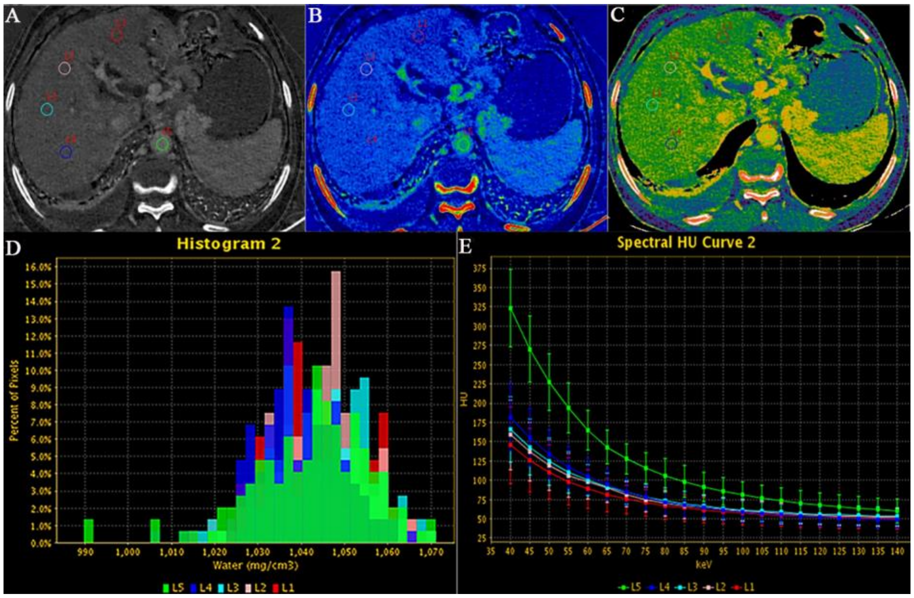

Fig.S1. (Progressive group) Dual-energy CT equilibrium phase imaging of the liver in a 58-year-old woman with HBV LC-AD. A-E respectively show the iodine (water) map, iodine (water) pseudo-color map, effective atomic number map, histogram, and slope of the energy spectrum curve, with iodine concentration (IC) =22.84, effective atomic number (Z) =8.94, energy spectrum curve (K_{140}) =1.76 and extracellular liver volume ($ECV_{IC\text{-}liver}$) =36.74.

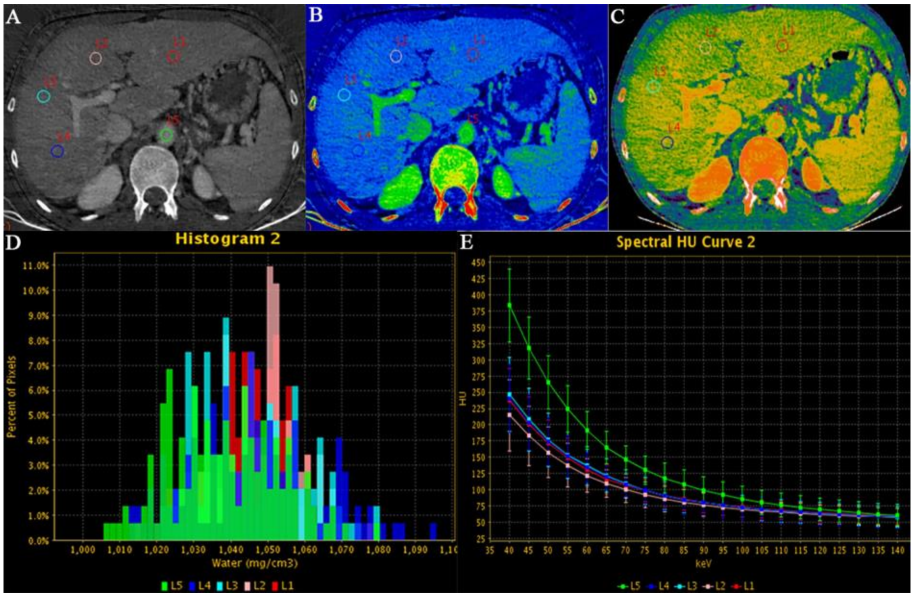

Fig.S2. (Stable group) Dual-energy CT equilibrium phase imaging of the liver in a 34-year-old man with HBV LC-AD. A-E respectively show the iodine (water) map, iodine (water) pseudo-color map, effective atomic number map, histogram, and slope of the energy spectrum curve, with iodine concentration (IC) =15.63, effective atomic number (Z) =8.52, energy spectrum curve (K_{140}) =1.21 and extracellular liver volume ($ECV_{IC\text{-}liver}$) =26.13.
